# Multiple sclerosis and palliative care - perceptions of severely affected multiple sclerosis patients and their health professionals: a qualitative study

**DOI:** 10.1186/1472-684X-13-11

**Published:** 2014-03-19

**Authors:** Heidrun Golla, Maren Galushko, Holger Pfaff, Raymond Voltz

**Affiliations:** 1 Department of Palliative Medicine, University Hospital of Cologne, Kerpener Strasse 62, 50924 Cologne, Germany; 2 Institute for Medical Sociology, Health Services Research and Rehabilitation Science (IMHR), Faculty of Human Science and Faculty of Medicine, University of Cologne, Eupener Strasse 129, 50933 Cologne,Germany; 3 Center for Integrated Oncology Cologne/Bonn, University Hospital of Cologne, 50924 Cologne, Germany; 4 Center for Clinical Trials, University Hospital of Cologne, 50924 Cologne, Germany

**Keywords:** Attitudes towards palliative care, Health professionals, Severely affected multiple sclerosis patients, Palliative care, Multiple sclerosis

## Abstract

**Background:**

In Germany, patients severely affected by Multiple Sclerosis (MS) do not routinely come into contact with palliative care, even if possibly beneficial. This study was aimed at investigating how severely affected MS patients and their health professionals perceive palliative care to determine how to better approach these patients in Germany about this topic.

**Methods:**

15 patients feeling severely affected by MS and 23 health professionals experienced with MS patients (3 social workers, 7 nurses, and 13 physicians) in both in-/outpatient and rural/ urban settings participated in this qualitative study in Germany. Semi-structured interviews (patients, health professionals) and focus groups (health professionals) were conducted, transcribed verbatim and analyzed via qualitative content analysis.

**Results:**

*MS patients* were mostly unfamiliar with the term “palliative care” or were aware of it only in relation to cancer and dying. They did not view it as relevant to themselves. *Health professionals* predominantly associated palliative care with dying cancer patients, if familiar with it at all. Most *physicians* doubted its relevance for neurological patients and denied MS as a cause of death. Nevertheless, most felt they already offered their patients sufficient palliative care, or thought that it could not meet MS patients’ complex needs. Most *nurses and social workers* recognized deficits in existing care structures and regarded palliative care as an opportunity for MS patients.

**Conclusion:**

MS patients’, and health professionals’ restricted, death-associated awareness of palliative care leads to discomfort, fear or rejection of this idea. Therefore, a defined concept of palliative care emphasizing opportunities for severely affected MS patients and considering early integration should be spread throughout the German MS community as an additional layer of support for this patient group.

## Background

Despite clear advances in research and treatment [1] Multiple Sclerosis (MS) remains a chronic, inflammatory, incurable disease of the central nervous system. Chronic progressive MS develops among 30-40% of patients within 10 to 15 years after diagnosis, with a significant proportion of patients having to cope with severe physical disability as well as psychological implications [2-4]. Approximately half of all MS patients die from MS itself or related complications, including accidents, severe falls, and suicide, evincing a culmination of severe psychological problems; approximately another third die from cardiac and vascular disease, cancer, infectious and respiratory diseases [5-7]. MS patients’ level of suffering from their “burden of symptoms” is comparable with that of palliative cancer patients [8], and MS patients and health professionals identify complex unmet needs which might be met by palliative care services [9-15]. Therefore, neuro-rehabilitation and palliation must play an important role in the management of MS patients beyond disease modifying treatments [2,11-14,16,17]. Presently, however, MS patients in Germany do not routinely come into contact with the palliative care approach, neither through their general providers nor via specialized palliative care services and institutions. This is possibly because it appears to be counterintuitive to many providers, in contrast to the UK’s pioneering role in implementing palliative care also for patients with neurological long term conditions [11-14,18-20].

Concepts designed to bring the palliative care approach to Germany’s MS patients must take into consideration the attitudes of both patients and associated health professionals. This qualitative study aimed to investigate how severely affected MS patients and health professionals in Germany understand palliative care, what they associate with this approach and if, or how, they feel palliative care might help these patients.

## Methods

### Design

Qualitative study of face-to-face cross-sectional, semi-structured interviews and focus groups with patients and health professionals analyzed via qualitative content analysis.

### Context and sample

Patients and health professionals were selected using a mix of purposeful and convenience strategies; this was done within a larger study frame on ‘unmet needs of severely affected MS patients’ in Germany [10,15]. All participants provided informed written consent. The Committee for Medical Research Ethics of the University of Cologne approved the study (IRB approval #06-191).

#### Health professionals

Physicians, nurses and social workers from a rural clinic specialized in MS and from 3 city neurology wards received invitations to participate in the study via post, email or telephone. An additional 60 Cologne city neurologists working in private practice and 88 GPs were contacted by mail. Ultimately 13 physicians, 7 nurses and 3 social workers agreed to participate in the study. Reasons for refusal cited lack of time, lack of remuneration for study participation or simply inexperience with MS [10].

#### Patients

A population of MS patients who reported feeling severely affected by their disease was recruited for this study. Health professionals in Cologne, Germany and surrounding areas (GPs, in- and outpatient neurologists, nurses, MS clinic team, city/rural area) were asked to help recruit MS patients by distributing leaflets about the study and by querying patients directly during visits to their doctor. For her interviews MG discussed participation with clinic patients during rounds. Of 22 patients approached for the study, 15 agreed to take part. Reasons for refusal were not assessed [15].

### Data collection

#### Health professionals

Semi-structured interviews were conducted with health professionals (2 social workers, 2 GPs, 5 neurologists), while others participated in a focus group (first group: 2 nurses and 1 social worker; second group: 6 neurologists in private practice; third group: 5 nurses). Interviews and focus groups were both conducted at the Department of Palliative medicine (University Hospital of Cologne) or at participants’ working place and lasted 30 - 90minutes (HG, MG). Socio-demographic information was recorded using a questionnaire completed by all health professionals following the interview/focus group [10].

#### Patients

Semi-structured face-to-face interviews (25 - 120 minutes) were conducted by MG at patients’ homes (4 patients) or on neurology wards (11 patients; short-term inpatient care, 1 of whom was normally in long-term nursing home care). Patients who so desired were allowed to have a relative present during their interview (5 instances). Each patient’s disease-specific information was collected via a short questionnaire including the EDSS (Expanded Disability Status Scale) [21] and a self-developed, non-validated scale (1-10) for rating the extent of feeling severely affected (“How severely affected are you?”, where 1 = not at all and 10 = totally severely affected) [15].

Our study emerged from efforts to understand the broader topic of unmet needs of severely affected MS patients in Germany which was investigated by using a mixed method design [10,15,22]. In this greater study frame on unmet needs of severely affected MS patients, pilot-tested interview guidelines for the different stakeholders were first developed in the qualitative study phase. Interviews and focus groups provided an opportunity to ask questions according to interview guidelines about unmet needs, feeling severely affected by MS and about palliative care [10,15]. The question of how severely affected MS patients and involved health professionals understand palliative care was singled out for this study, while we also hoped to discern what they associate with this approach and if, or how, they feel palliative care might help these patients. So, for the results presented here only those questions of the interview guidelines asking about participants’ associations with, and attitudes towards palliative care/hospice were relevant. Examples of questions on this topic for health professionals were: “In your view, what does palliative care/hospice mean? Under which conditions could you imagine using palliative care services for your MS patients?” and for patients: “What ideas do you have about palliative care/hospice? Under which conditions can you imagine to use palliative care services for yourself?” No definition of palliative care was offered to interviewees as we were interested in their unbiased ideas, associations and attitudes towards palliative care in general and in context with MS patients. Investigators’ basic definition for the concept of palliative care conforms to the 2002 WHO statement:

“Palliative care is an approach that improves the quality of life of patients and their families facing the problem associated with life-threatening illness, through the prevention and relief of suffering by means of early identification and impeccable assessment and treatment of pain and other problems, physical, psychosocial and spiritual” [23]. All investigators were well informed and up-to-date regarding the developing structures of palliative care, both general and specialized, in Germany.

Audio recordings were made of the semi-structured interviews and focus groups, and then transcribed verbatim; these were not returned to participants. Field notes were also made (HG, MG). MG (sociologist) and HG (MD) worked as researchers at the Department of Palliative medicine and were experienced in interviewing palliative care (MG, HG) and neurological (HG) patients as well as health professionals (MG).

### Analysis

Interviews and focus groups were first analyzed for evolving categories of unmet needs as previously described [10,15]. This paper concentrates on the analysis of attitudes towards palliative care held by health professionals and severely affected MS patients:

All transcripts of interviews and focus groups were separately, inductively coded with open codes by one of the interviewers. Thematic units concerning attitudes towards palliative care were analyzed and categorized using the method of constant comparison (HG, MG). MG verified evolving categories and issues found by HG and vice versa. The raw data and related codes were then multi-professionally discussed amongst all authors (HG, MG, HP, RV) [24]. Respondents’ implicit views were then included into the coding process. The constant comparison of the codes for explicit and implicit views of all cases (focus groups and interviews) were searched for minimal/maximal contrasts (HG, MG) [25,26]. This comparison resulted in ongoing refinement from first codes to preliminary categories and then to categories on a higher abstraction level that could be applied to all data. MG checked the coding process and plausibility of evolving preliminary categories found by HG, and vice versa. For focus groups we also considered the group interaction and the sequence of topics [27-29]. This study conforms to COREQ guidelines for reporting qualitative research [30].

## Results

### Sample

Characteristics of the samples are summarized in Tables 1 and 2.

**Table 1:** Characteristics of MS patients interviewed

**Form of disease (Number in brackets)**	**Gender** * **(Number in brackets)**	**Age [years]**	**Subjective affectedness**	**EDSS**	**Family situation (Number in brackets)**	**Occupation state (Number in brackets)**
Relapsing-remitting (5)	Female (3)	Range: 34 - 54	Range: 5 - 8	Range: 5 – 6,5	Married (4)	Full-time working (2)
	Male (2)	Mean: 40,4	Mean: 6,6	Mean: 6	Unmarried (1)	Retired (2)
				(1 Unknown)		Unemployed (1)
Primary progressive (4)	Female (2)	Range: 23 - 73	Range: 6 - 10	Range: 3 - 9	Married (2)	Full-time working (1)
	Male (2)	Mean: 55	Mean: 7,3	Mean: 6,4	Unmarried (1)	Retired (3)
					Widowed (1)	
Secondary progressive (3)	Female (2)	Range: 39 - 56	Range: 6 - 8	Range: 6 - 8	Married (2)	Full-time working (1)
	Male (1)	Mean: 45	Mean: 7,3	Mean: 7	Unmarried (1)	Retired (2)
Unknown (3)	Female (3)	Range: 40 - 55	Range: 4 - 10	Range: 6,5 – 8,5	Married (1)	Retired (3)
		Mean: 49,7	Mean: 6	Mean: 7,5	Unmarried (1)	
				(1 Unknown)	Divorced (1)	

*To guarantee anonymity, we use the male form only in the text.

**Table 2:** Characteristics of health professionals interviewed

**Profession (Number in brackets)**	**Working place (Number in brackets)**	**Participation in**	**Professional experience [years]**	**Experience with MS [years]**	**Gender** * **(Number in brackets)**	**Age [years]**
Nurses (7)	MS outpatients (2)	First focus group	Range: 15 - 28	Range: 0,5 - 20	Female (6)	Range: 35 - 57
	Specialized MS clinic, inpatient (5)	Third focus group	Mean: 23 (1 Missing data)	Mean: 11,4	Male (1)	Mean: 46
Social workers (3)	Clinic for neurology and psychiatry (1)	First focus group	Range: 28 - 30	Range: 10 - 30	Female (2)	Range: 47 - 52
	Specialized MS clinic (1)	Interview	Mean: 29	Mean: 22,3	Male (1)	Mean: 49,3
	Palliative care unit (1)	Interview				
Physicians (13)	Neurologists in own practice (6)	Second focus group	Range: 6,5 – 30	Range: 6,5 – 30	Female (5)	Range: 35 - 59
	General practitioners in own practice (2), one of them additionally nursing home doctor	With each interview	Mean: 18,7 (1 Missing data)	Mean: 16,9	Male (8)	Mean: 46,8 (1 Missing data)
	Clinic neurologists	With each interview				
	- Specialized MS clinic (2)					
	- General neurology clinic (3)					

*To guarantee anonymity, we use the male form only in the text.

### Categories

**Figure 1:**
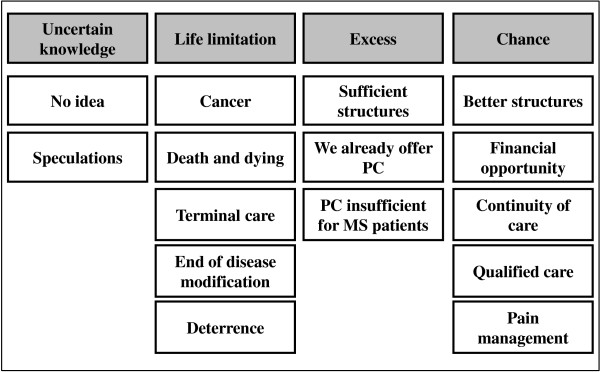
Associations with palliative care (PC) for MS: System of categories evolving from all stakeholders, MS patients as well as health professionals.

According to findings of this study, associations with palliative care by severely affected MS patients and health professionals caring for such patients can be classified into four main categories: ‘uncertain knowledge’, ‘life limitation’, ‘excess’ and ‘chance’ all of which could be further divided into subcategories (Figure 1).

**Figure 2:**
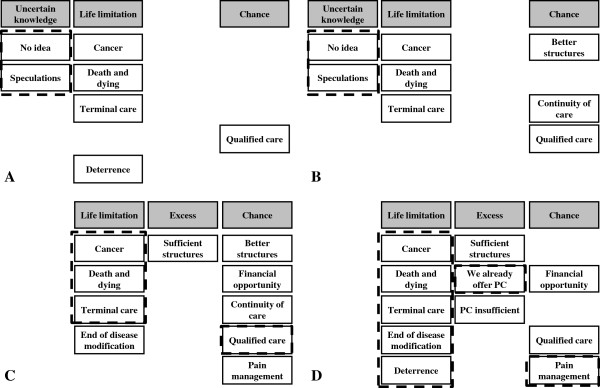
Associations with palliative care (PC) for MS: System of categories evolving from A Patients (n = 13), B Nurses (n = 7), C Social workers (n = 3) and D Physicians (n = 13); dashed lines in all boxes (A-D) for a special focus: A Patients and B Nurses mostly have no, or vague ideas of palliative care; C Social workers agree that palliative care offers qualified care for terminally ill cancer patients; D Physicians concentrate on palliative care as qualified care for terminally ill cancer patients.

Figure 2A-D depicts an itemization for the different stakeholders’ attitudes (patients, nurses, social workers and physicians); followed by a detailed description of each stakeholder group’s view below.

### MS patients

Figure 2A summarizes the categories of patients’ associations with palliative care. Two interviews were prematurely terminated due to patient exhaustion.

#### Main category uncertain knowledge

Of the remaining 13 patients, six had no idea what palliative care meant (“foreign word”) (*subcategory no idea*) and seven patients had a rather vague notion (*subcategory speculations*): One patient speculated that palliative care meant “acute treatment of cancer patients”; for another it was support for chronically ill patients, while others guessed that it was “alternative”, “traditional”, or “ancient” medicine. Patients interviewed had heard of hospice slightly more often than palliative care, but could not differentiate between the terms or were rather uncertain in this regard. While some patients thought hospice was a hospital, others wondered whether adequate medical care was guaranteed there at all, and one patient thought that access to hospice would be difficult.

#### Main category life limitation

Those who had heard of palliative care mainly regarded it in the context of hospice, critical illness and especially cancer (*subcategory cancer*), or dying (*subcategory death and dying*), and characterized it as terminal care or as help to die (*subcategory terminal care*). Patients could not imagine palliative care for themselves. They felt this approach would come too early in their life or they could not associate it with MS and their own situation at all.

If I was really **seriously** ill and and really unable to do absolutely anything alone, **then** I would consider this. […] However, otherwise […] whether this absolutely has to be, to think, about this now already […] when someone is incurably ill with cancer, for example, then that is the time for palliative care that is essentially …end of life care […]

Interview with patient, secondary progressive MS (IntGa1 ll. 1593-1612)

Some were even afraid of hospice and considered life in a hospice as a helpless life no longer worth living (*subcategory deterrence*).

[…] Hospice is actually terrifying […] linked for me with being absolutely helpless, the last step, and actually just vegetating […] keep it at a distance. Best not to experience this.

Interview with patient, relapsing-remitting MS (Intga8 ll. 1069-1086)

#### Main category chance

In the context of terminal care for cancer patients, MS patients principally thought of palliative care as a positive approach. Palliative care or hospice was predominately well-respected. The expressed view was that it stands for qualified care for dying cancer patients encompassing pain treatment and support for both the dying and their relatives (*subcategory qualified care*).

### Health professionals – Nurses

Figure 2B summarizes the categories of nurses’ associations with palliative care.

#### Main category uncertain knowledge

Three of the seven participating nurses (Table 2) had no idea of what palliative care was; two of these individuals work with MS outpatients and one with MS patients in a specialized MS clinic (*subcategory no idea*). The other four who work in a specialized MS clinic had vague (e.g. “alternative” medicine) and limited ideas of palliative care; making associations such as company, palliation and support (*subcategory speculations*). They confused the terms palliative care and hospice. The nurses knew of the existence of hospice services but had difficulty imagining their tasks.

#### Main category life limitation

Hospice and palliative care were linked with “last stop” and “final situation” (*subcategory death and dying*). It was appreciated as dignified terminal care (“kind dying”) (*subcategory terminal care*) especially for dying cancer patients (*subcategory cancer*).

#### Main category chance

Palliative care was principally linked with qualified care and the interviewed nurses thought that such care would also be worthwhile for severely affected MS patients (*subcategory qualified care*). Moreover, the nurses have made the experience that nursing homes are not prepared for the complex needs of MS patients, leading to care deficits. They had the expectation that the palliative approach could offer a better alternative (*subcategory better structures*). In particular, they hoped that palliative care could offer MS patients a continuous contact person who accompanies, offers answers to questions and points out new opportunities (*subcategory continuity of care*).

### Health professionals - Social Workers

Figure 2C summarizes the categories of social workers’ associations with palliative care.

The three social workers (Table 2) disagreed on whether, how and to what degree palliative care should be available for MS patients.

#### Main category life limitation

The social worker at a specialized MS clinic thus far knew palliative care only in connection with cancer patients (*subcategory cancer*) but held the view that MS patients could also benefit from it. The palliative care unit social worker regarded it as a worthwhile holistic approach irrespective of diagnosis but recognized that it was primarily offered to dying cancer patients (*subcategory cancer*, *subcategory death and dying*). The neurology/psychiatry social worker acknowledged the value of palliative care only for cancer patients (*subcategory cancer*). His view was that cancer palliation should start once tumor therapy had been exhausted (*subcategory end of disease modification*), and a place for dying should be organized (*subcategory death and dying*); regarding hospices as institutions where patients and relatives would not be left alone at the point of death and dying (*subcategory terminal care*).

#### Main category excess

The neurology/psychiatry social worker was convinced that with sufficient advance planning MS patients would not need palliative care. He regarded nursing homes as appropriate institutions for MS patients only if these possess the requisite knowledge and experience with MS patients. His opinion was that this is the most decisive factor as to whether MS patients would receive appropriate care, and that having additional knowledge about palliative care would not improve MS patients’ care in his view. He also expressed the view that palliative care units could not offer more to MS patients than good neurological units, as such neurological units are specialized in care for MS patients and thus possess sufficient knowledge required to meet MS patients’ concerns (*subcategory sufficient structures*). The main arguments against palliative care for MS patients, in his view, were the long-lasting and unclear disease trajectory, and that as he understood it, the typical burdensome symptoms of MS patients could not be palliated as effectively as the pain of cancer patients, for example.

These chronic illnesses go on a **long** time, many years, so I wouldn’t necessarily see palliative care as sensible, […] I can’t see any real sense behind this. […] apart from, maybe, from a temporary respite […] so I, for me, that is rather more an excess of care I would say.

Social worker, clinic for neurology and psychiatry, focus group of nurses and social worker (fg1ga ll. 1739-1757)

#### Main category chance

The specialized MS clinic social worker regarded palliative care as a worthwhile opportunity also for MS patients: He expected palliative care to offer pain management (*subcategory pain management*); individual, intensive, perhaps even one-on-one care which considered everyday life wishes, support in accepting MS as well as preparation for dying (*subcategory qualified care*). He stressed the potential financial opportunities which may open to support MS patients should they be considered for palliative care and special funding as are cancer patients (*subcategory financial opportunity*). He presumed the necessity for long-lasting continuous support because of the lengthy, unpredictable disease trajectory (*subcategory continuity of care*).

And when […] palliative care makes it possible to have access to other resources, […] financial sources, and if we could attain this for the most critically ill MS patients, umm, there where there is no money to meet the needs, and close the gaps in the care, then that would also by all means be an important point.

Interview with social worker, specialized MS clinic (gaso1 ll. 109-128)

The palliative care unit social worker stressed that in his understanding, independent of diagnosis, the individual patient’s situation is the decisive factor as to whether palliative care, in all its forms, with its extensive opportunities, is utilized or not. His opinion was that a stay on a palliative care unit, for example, could re-energize both the MS patient and (informal) caregivers, providing the basis for a return to home care (*subcategory better structures*). He defined palliative care as a holistic approach covering medical, nursing and psychosocial aspects using a multidisciplinary team who focus on patients’ positive resources even in the face of death (*subcategory better structures*).

So I think it’s really important that […] palliative care […] **is** open to all areas of illness. And umm, […] MS patients with severe symptoms should also have the opportunity, to have intensive care on the palliative ward as their illness **is** incurable, it continues progressing […]

Interview with social worker, palliative care unit (goso2 ll. 496-503)

### Health professionals – Physicians

Figure 2D summarizes the categories of physicians’ associations with palliative care.

All 13 physicians queried, independent of specialty, working place, or position (Table 2) had some idea of palliative care.

#### Main category life limitation

Most regarded palliative care as a field of palliation instead of curing, with predominant or even complete substitution of etiological treatment. The three general neurology clinic neurologists, for example, indicated they could envision a palliative care approach for MS patients once they were no longer being treated with immunosuppressive or immunomodulatory medicines (*subcategory end of disease modification*). Nevertheless, most of the physicians interviewed only minimally accepted such palliative care approaches for neurological diseases like MS, if at all, and then in advanced and terminal cases only (*subcategory terminal care*).

[…] that there […] could also be palliative care for persons suffering from MS is perhaps a relatively wild idea. […] different from, for example, cancer patients, where one more likely intuitively believes that also palliative care might make sense, is rather unusual for those suffering from MS.

Interview with clinic neurologist, general neurology clinic (ar1ga ll. 134-143)

These physicians assessed the disease trajectory of MS as less clear than that of cancer. They also stated that MS patients would not die from their disease, regarding this as the main reason for not pursuing a palliative care approach with neurological (MS, Parkinson’s etc.) patients. They emphasized that palliative care was automatically associated with death and dying (*subcategory death and dying*) resulting in both physicians and patients having reservations (*subcategory deterrence*). In most cases neurologists would lead their MS patients to believe that their life expectancy would not be shortened by MS. Particularly neurologists in private practice viewed the main task of palliative care in managing terminal cases as guaranteeing a sort of “kind dying,” at home, if possible (*subcategory terminal care*). Such terminal situations were, however, consistently assumed to be solely in the context of cancer patients, not neurological patients (*subcategory cancer*).

[…] generally, as a rule our neurological patients DON’T die from these neurological disorders - but they die from other deficits, umm which develop from these disorders, […] MS patients or Parkinson’s or whatever, umm their lifespan is not limited by this illness […].

Neurologist in focus group of neurologists, all in own practice (fg2ga ll. 1277-1288)

#### Main category excess

Except for one, all general practitioners and neurologists in private practice queried felt convinced that they already offered their MS patients sufficient palliative care (*subcategory we already offer PC*) when utilizing their broader, more general definition of palliative care such as “helping to live better with the illness”.

Depending on how one defines it well, yes. Everything that no longer heals is palliative, err…, certainly however that is not meant […] all that we do, is umm to live better with the illness and somehow accompany, yes. […] Actually, well we do this already the entire time, right?

Neurologist in focus group of neurologists, all in own practice (fg2ga ll. 1390-1412)

The two specialized MS clinic neurologists principally regard MS patients as palliative care patients since they suffer from an incurable, chronic disease. These physicians regarded their work at the specialized MS clinic as palliative work, managing the complex needs of MS patients (*subcategory sufficient structures*) and doubted that palliative care units were able to meet the special needs of MS patients due to their lacking the broad spectrum of therapies currently available which have been specialized for these patients (*subcategory PC insufficient*).

[…] also not on the palliative ward, as no therapies take place there, there is no physiotherapy, which is of course very **important** for treating spasticity, particularly when they are lying down. That is the point where palliative care is incompatible with MS.

Interview with clinic neurologist, specialized MS clinic (galar5 ll. 351-356)

#### Main category chance

Most of the physicians interviewed regarded palliative care as an approach aimed at achieving the best available quality of life for the patient in the time remaining. They emphasized qualified care, especially with respect to end of life (*subcategory qualified care*) and professional pain treatment (*subcategory pain management*). In the view of the neurologists at the specialized MS clinic, palliative care could aid MS patients with greater financial support as required for hospital stays long enough to improve certain symptoms, which often involves weeks or even months (*subcategory financial opportunity*).

## Discussion

This study specifically documents perceptions of palliative care of severely affected MS patients and their healthcare professionals in Germany. The results give first insights into why severely affected MS patients in Germany only have sparse access to palliative care in all its forms, including hospice care, and aid in generating hypotheses about how to better approach this situation.

Of all those interviewed, the MS patients had the fewest associations with palliative care and they had difficulties in differentiating between hospice and palliative care. One possible explanation for this lack of awareness might be that advanced MS patients might have, over time, become adjusted to living with their disabilities and thus, as becomes clear in this study, might not view their condition as ’terminal’ and in need of palliative care. While in general, patients depend on advice from their health professionals, it is the nurses who are often closest to patients and of great importance to them [31], and of all the health professionals interviewed it was the nurses who could at least imagine what was meant by palliative care. Physicians and social workers had the broadest spectrum of associations with palliative care but they predominately focused on ‘life limitation’ or on the idea that ‘we already offer palliative care’. However, it was apparent that they were unsure of what palliative care exactly encompasses, who offers it and whether it would be useful for long-term neurological conditions like MS. These physicians and social workers made no distinction between general and specialized palliative care and never mentioned these terms. As patients and nurses are strongly influenced by physicians’ opinions it is no wonder that neither of these groups are well informed about palliative care.

In fact, the fears surrounding its close association with death were far more recognized, along with those related to questioning the capability and expertise of extant care structures, than were overall opportunities for utilizing palliative care for MS patients.

In this study one important reason why palliative care was rejected for MS patients was the overwhelming opinion that palliative care would be meaningful only for terminal diseases or for stages typical of various cancers. Indeed the illness trajectory of MS and the terminal care phase are less distinct [32,33] and doubts exist about whether patients actually truly die from MS [34]. However, death clearly plays a role in MS, as MS patients could die from their disease or associated complications [5-7]. As acknowledged by participants in this study, the handling of death and dying, ostensibly the core focus of palliative care [32,35], might also play a role in the management of MS patients [36], so MS and palliative care are not contradictory, even if regarded in light of death and dying. We also know that severely affected MS patients are interested in communicating with their doctors about end of life issues [22] but doctors hardly ever address this topic with their MS patients although it might be beneficial both for patients and their families [37].

Another critical point brought up by the participants in our study was that they predominately associated palliative care with accompanying dying cancer patients. In contrast to cancer, the end-of-life phase in MS would be difficult to detect and symptom relief would play a comparatively minor role with MS patients. However, severely affected MS patients are every bit as afflicted as cancer patients in terms of the characteristics of the symptoms, and needs assessment of (severely) affected MS patients revealed unmet needs in those areas which could possibly be met by a palliative concept [8-11,15]. Nevertheless, compared with cancer patients, non-cancer patients, such as MS patients in Germany, do not have equal access to (specialized) palliative care and hospice services [32,34,38]; e.g. between 1993 and 2010 only 2.5% of patients whose deaths were attributed to MS died in hospice (UK) [39]. Reasons for less access to these services are that MS patients or their providers –as in our study– are rarely aware of such opportunities [34] or an advance care plan is lacking [37,40]. Our study indicates that both patients’ and health professionals’ fearful attitudes towards palliative care may also play a role in this.

Such fears might potentially emerge from the restricted and even vague awareness of palliative care exposed in this study, and these vague ideas about palliative care correspond to the various, partially discordant definitions of ‘palliative care’ and ‘the palliative care patient’, especially non-cancer palliative care patients [41-43]. A standardized and consistent definition and care concept of palliative care offering a reliable understanding of palliative care philosophy, aims, structures and activities could also aid in the German MS community discovering and appreciating previously unforeseen or overlooked opportunities for MS patients and their caregivers. Fears and doubts in the context of palliative care could then be resolved, making renaming ‘palliative care’ ‘supportive’ or ‘comfort care’ [35,42,44,45] unnecessary as well.

In the UK there is now a document available addressing how palliative care in all its forms could improve care of patients with long-term neurological conditions: “End of life care in long term neurological conditions – a framework for implementation” (NEoLCP) [20]. Pioneering studies in the UK have also successfully utilized specialized palliative care services for MS [12-14] or for neurodegenerative conditions [46].

The response ‘We already offer palliative care’, encapsulates a serious concern brought up by physicians in our study. This reply represents the idea that institutionalized palliative care might question the capability and expertise of existing care structures. Such concerns generally arise in the debate on how specialized outpatient palliative care could contribute to general (palliative) care without displacing existing structures [47]. While the palliative care approach alone cannot alleviate the complex needs of MS patients at large, nor in Germany, as related to our study, a common care concept for MS patients is needed which encompasses the expertise of general care providers as well as of palliative, neuro-rehabilitative and MS specialists. The MS community has ample knowledge of the disease, its progression, disease-specific treatment, needs and complications; including where special multidisciplinary rehabilitation programs can often stabilize or improve patients’ functions. The palliative care community is primarily concerned with patient quality of life, autonomy in end of life decisions, palliation of burdensome symptoms and accompanying MS patients and their families through the various in- and outpatient sectors with assistance from multidisciplinary teams. General care providers are continuously involved at the ground level and are familiar with the typical problems of everyday life. These diverse skills are complementary and valuable for MS patients throughout the different stages of the disease. In contrast to Germany, the UK has already been developing such a multidisciplinary approach for long-term neurological conditions like MS, Parkinson’s, motor neuron diseases and Huntington’s disease and this is now spreading. The NEoLCP [20] offers different health professionals in different settings a wide range of practice guidance for the palliative care of neurological patients. Germany must first reach agreement among representatives of general, MS, neuro-rehabilitative and palliative care as to who is responsible, when and for what part of an MS patient’s care. Agreement is needed on when palliative care should be offered to MS patients, and specifically agreement about when end of life issues should be considered with these patients. Indicators have been determined and include swallowing problems, aspiration pneumonia, recurrent infections or marked decline in physical status [36]. In defined tumor entities early integrated palliative care has been shown to improve quality of life and even lead to significantly longer survival [48]. It is highly conceivable that MS patients might also profit from such an early integration of palliative care. If possible treatment restrictions are already discussed during the early stages of a disease, then quality of life might improve in later stages of chronic neurological diseases [37].

How can palliative care be brought to MS patients, and in our case Germany’s MS patients, on a widespread basis and meanwhile dispel common fears associated with it? Initial, low-threshold palliative care contact could be undertaken by general providers (e.g. GPs, neurologists), as emphasized by the physicians surveyed for our study. Ensuring early access to general palliative care requires that appropriate palliative care training be widely offered for multidisciplinary MS caregivers. In complex situations specialized in- or outpatient palliative care services with essential knowledge about MS might be an asset in helping to alleviate suffering, as proven in the UK where specialized outpatient palliative care services for MS or neurodegenerative conditions have been successfully utilized [12-14,46]. The UK studies explicitly make the crucial delineation that their palliative services were complementary, and not a substitute for existing services [12-14,46], both confirming and emphasizing the importance of coexistent providers.

### Limitations and strengths of study

As interviews and focus groups could not be conducted to theoretical saturation, the study results cannot be generalized. There were barriers prohibiting interviews with advanced MS patients and similarly, communication disabilities, cognitive impairments, depression or fatigue made it problematic to conduct some interviews. Recruiting difficulties with health professionals meant that the groups interviewed were not equal in number; physicians (and neurologists among them) represented the largest group. However, the qualitative approach of this study made it possible to combine narrative elements with semi-structured questions. The interview guide was used flexibly, enabling the interviewees to elaborate their perspectives. More detail on various attitudes was obtained via this approach. A rigid format of a structured interview or a questionnaire would not have allowed us to gather this spectrum of information.

## Conclusion

Severely affected MS patients and health professionals in Germany may have a vague and incomplete perception of palliative care, leading to misunderstandings, discomfort or even fear and rejection. A consistent palliative care concept with a view towards early integration should be introduced and made public within the MS community and among general providers in Germany so as to offer, in a combined approach, an additional layer of support for severely affected MS patients in the future.

## Abbreviations

MS: Multiple sclerosis; PC: Palliative care; EDSS: Expanded disability status score.

## Competing interests

The authors declare that they have no competing interests.

## Authors’ contributions

RV, HP, MG conceptualized the study. RV obtained funding. HG and MG were responsible for the overall study coordination including recruitment, data collection and data analysis. HG wrote the manuscript and MG, RV and HP commented on it and made further suggestions until the final form was reached which was approved by all authors.

## Pre-publication history

The pre-publication history for this paper can be accessed here:

http://www.biomedcentral.com/1472-684X/13/11/prepub
